# Updates to Protex for Simulating Proton Transfers in an Ionic Liquid

**DOI:** 10.1021/acs.jpcb.3c07356

**Published:** 2024-04-01

**Authors:** Márta Gődény, Florian Joerg, Maximilian P.-P. Kovar, Christian Schröder

**Affiliations:** † Faculty of Chemistry, Department of Computational Biological Chemistry, University of Vienna, Währinger Straße 17, Vienna 1090, Austria; ‡ University of Vienna, Vienna Doctoral School in Chemistry (DoSChem), Währinger Straße 42, Vienna 1090, Austria

## Abstract

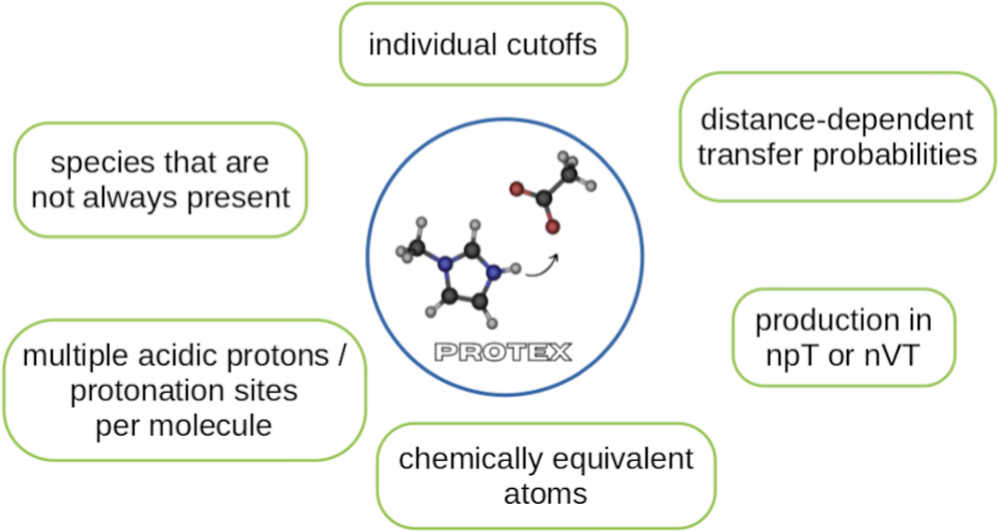

The Python-based program Protex was initially developed for simulating proton transfers in a pure protic ionic liquid via polarizable molecular dynamics simulations. This method employs a single topology approach wherein deprotonated species retain a dummy atom, which is transformed into a real hydrogen atom during the protonation process. In this work, we extended Protex to include more intricate systems and to facilitate the simulation of the Grotthuss mechanism to enhance alignment with the empirical findings. The handling of proton transfer events within Protex was further refined for increased flexibility. In the original model, each deprotonated molecule contained a single dummy atom connected to the hydrogen acceptor atom. This model posed limitations for molecules with multiple atoms that could undergo protonation. To mitigate this issue, Protex was extended to execute a proton transfer when one of these potential atoms was within a suitable proximity for the transfer event. For the purpose of maintaining simplicity, Protex continues to utilize only a single dummy atom per deprotonated molecule. Another new feature pertains to the determination of the eligibility for a proton transfer event. A range of acceptable distances can now be defined within which the transfer probability is gradually turned off. These modifications allow for a more nuanced approach to simulating proton transfer events, offering greater accuracy and control of the modeling process.

## Introduction

Ionic liquids are salts typically characterized by a low melting point, often below room temperature. Protic ionic liquids (PILs) are a subclass that is composed of a Brønsted acid and a Brønsted base, thereby enabling reversible proton transfers. This characteristic confers high mobility to protons, culminating in enhanced conductivity. The conductivity is particularly increased in scenarios where the Grotthuss mechanism^1^ of proton transport is feasible.^2−5^ Due to this attribute of superior conductivity, PILs have become the subject of extensive scientific research, with a focus on their potential application as electrolytes in battery technologies.^5,6^

Molecular dynamics (MD) simulations constitute an effective tool for examining the transport properties of ionic liquids. Moreover, collective properties, such as viscosity and conductivity, can be inferred from their respective trajectories. Despite the application of classical MD simulations to PILs,^7−13^ the inability to model proton transfers poses a significant constraint on the insights obtainable from this approach.

In principle, proton transfers can be modeled by a hybrid quantum-mechanical (QM)/classical mechanics approach.^14^ The reacting partners are described quantum-mechanically, and the surrounding molecules acting as solvents are handled by MD. However, the simulation system is strictly divided into the quantum and classical parts. This not only limits the proton transfer to one pair in the complete simulation box but also restricts the overall simulation period to short times. Empirical valence bond theory^15^ uses the different quantum states of the reactants and products to get the potential energy surface and hence the forces for MD simulations. Here, the system is not strictly divided into quantum and classical parts, but still the computational effort is quite high, particularly when considering hundreds of molecules to transfer protons. Contrarily, reactive force fields,^16^ such as ReaxFF,^17^ provide the capacity for continuous bond formation and breaking, thus circumventing the demand for computationally expensive quantum mechanics calculations. ReaxFF is a sophisticated force field with many parameters, necessitating an expansive training set to encompass the pertinent chemical phase space.^17^ This includes parameters for bond and angle stretches, activation and reaction energies, equation of state, surface energies, and numerous other elements. Although the application of all these methods has the potential to significantly increase the understanding of proton transfer in PILs but at a high computational cost that prevents the calculation of viscosity and conductivity. To the best of our knowledge, empirical valence bond theory and reactive force fields have not been employed to simulate PILs yet.

An alternative methodology is a constant pH simulation,^18−21^ originally developed for the simulation of proton transfers within proteins. Predominantly, these techniques involve alchemical approaches that utilize different λ-states, transitioning from λ = 0, representing a deprotonated state, to λ = 1, indicating a protonated state.^18−20^ However, these simulations often lack explicit protons. Instead, each protonation site is associated with a proton bath. This approach imposes a constraint on the number of viable protonation sites due to the requirement of adequate distancing between them to prevent mutual influence.^18^ Consequently, these methods prove to be inapplicable for PILs, where several hundred molecules hold potential for protonation or deprotonation, further highlighting the necessity for flexible and adaptable simulation techniques. Additionally, constant pH simulations cannot follow proton hopping from one species to another to increase the conductivity via the Grotthus mechanism.

**Figure 1:**
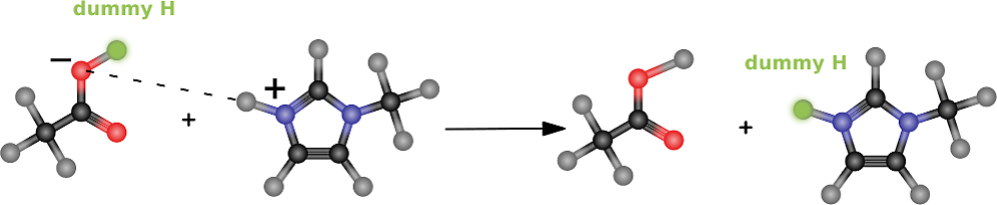
Proton transfer between Im_1_H^+^ and OAc^–^ in a single topology approach. Dummy atoms are shown in green, and the dashed line shows the distance that has to be smaller than the allowed cutoff radius for a successful transfer.

The Python-based program Protex^22^ can be used with the MD package OpenMM and adopts a single topology approach to surmount the aforementioned limitations. In this approach, deprotonated molecules retain a dummy atom with zero charge and Lennard-Jones parameters. This dummy hydrogen can transform into a real hydrogen atom upon protonation, simultaneously converting the real proton of the donor to a dummy proton. This is elucidated in Figure 1. This mechanism ensures a one-to-one correlation between the deprotonated and the associated protonated species, enabling a smooth transition of their force field parameters upon protonation as well as charge neutrality of the simulation box at all times. Consequently, a proton transfer with only two λ-states is facilitated. Although Protex can theoretically incorporate intermediary λ-states, their use is typically not recommended for the computation of dynamic properties such as diffusion or conductivity, as each intermediary step involves interactions with nonphysical species. However, intermediary steps could be utilized for properties solely dependent on the initial and final states, such as free energy differences.^22^ The free energy Δ*G* is an important property to estimate *pK*_a_-values computationally or compute their change in a particular solvent^23−25^
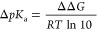
1

These changes directly facilitate or hinder proton transfers. The seamless interoperation with free energy calculating programs, like transformato,^26,27^ is also a strong benefit of Protex. In transformato, the atoms are distinguished into common core and dummy regions. For example, in Figure 1, the cationic common core region consists of the imidazolium ring. The anionic common core is the carboxylate group. The dummy regions in transformato are ring hydrogens and the methyl groups of the cations and anions. The change in *pK*_a_ when a methyl group is replaced by an ethyl group can be performed without computing the complete free energy cycle again.

The original Protex program^22^ periodically interrupts the production of the trajectory at designated proton exchange intervals (*pxi*) to conduct a list of acidic protons within a predefined proton exchange radius (*pxr*) from an acceptor. Subsequently, a transfer is executed with a predetermined probability for each specific donor–acceptor pair on that list.^28^ These transfer events trigger notable modifications in the force field parameters of the newly formed molecules, particularly affecting the partial charges of all constituent atoms.^22^ Under conventional circumstances, such alterations could considerably disrupt the stability of trajectory production. However, implementing polarizable forces mitigates this potential issue by smoothing the transient Coulomb energy. Therefore, Protex provides a robust and reliable platform for simulating complex proton exchange phenomena while accommodating significant changes in molecular properties.

## Methods

### Investigated Systems

**Figure 2:**
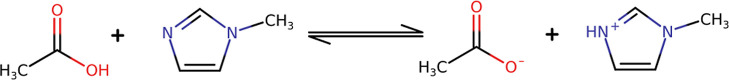
Equilibrium between the charged and neutral species in the 1-methylimidazolium acetate system.

Protex was originally developed for the pure “pseudoprotic” ionic liquid 1-methylimidazolium (Im_1_H^+^) acetate (OAc^–^), which is in equilibrium with the neutral species 1-methylimidazole (Im_1_) and acetic acid (HOAc), as shown in Figure 2. This equilibrium had been shown to lie at around 30% charged and 70% neutral species.^8^ The proton transfer probabilities were derived from one-dimensional QM scans and then checked with a Markov chain model to keep the ratio between charged and neutral species.^28^

For a comprehensive examination of proton hopping phenomena, the experimenter incorporated photoacid 8-hydroxy-1,3,6-pyrenetrisulfonic acid HPTSH^3–^. Upon laser excitation, this photoacid experienced a decrease in its *pK*_a_ value from 7.4 to 1.3, subsequently releasing a proton into the liquid medium. A second laser IR pulse was employed to monitor the vibrational spectrum and detect the absorption of the proton by the acetate molecules. Regrettably, the photoacid could not be dissolved in the pure PIL, necessitating the introduction of significant quantities of methanol into the mixture to avoid precipitation of the photoacid.

Experimental conditions were emulated by configuring systems composed of 1 m of the PIL and 60 mm HPTSH^3–^, dissolved in methanol (MeOH). Additional Im_1_H^+^ cations were employed to neutralize the charge of HPTSH^3–^, thereby maintaining a net charge of 0 e within the simulation box. The precise quantity of molecules within 40 and 70 Å boxes is detailed in Table 1.

**Table 1:** Composition of the Systems

	ref (8)	pure IL	pure IL	pure IL	ions	neutral	small	large
box	50 Å	50 Å	50 Å	50 Å	50 Å	50 Å	40 Å	70 Å
replicas	5	6	3	3	3	3	3	6
period	50 ns	50 ns	50 ns	50 ns	50 ns	50 ns	50 ns	50 ns
ensemble	nVT	nVT	nVT	npT	npT	npT	nVT	nVT
*pxr* variation	no	no	no	no	no	no	yes	no
*pxi* variation	no	no	no	no	no	no	yes	no
probability correction	yes	yes	yes	no	no	no	yes	yes
*c* variation	no	no	yes	no	no	no	no	no
species								
Im_1_H^+^	150	150	150	150	500	0	26	147
OAc^–^	150	150	150	150	500	0	17	93
Im_1_	350	350	350	350	0	500	40	217
HOAc	350	350	350	350	0	500	40	217
HPTSH^3–^	0	0	0	0	0	0	3	18
MeOH	0	0	0	0	0	0	702	3765

Smaller boxes served to scrutinize the impact of varying the proton exchange radius (*pxr*) between 1.55 and 1.62 Å, and the interval between proton transfers (*pxi*) ranging from 0.5 to 5 ps. Three replicas of each *pxi*/*pxr* combination were executed independently. The replicas of the larger box were simulated using standard parameters, specifically a proton exchange radius of 1.55 Å and an interval of 7 ps.

To systematically investigate the effects of the updates on Protex, the line of simulations carried out by Joerg et al.^8^ on systems consisting of pure PIL was also continued. In addition to the 30/70% ionic to neutral ratio, systems starting from 100% neutral and 100% ionic species were also set up to try to validate the Markov chain model.^28^ Production in these systems had to be performed in the npT ensemble, since the density and, thus, the box size depend on the degree of ionization.

We also examined the impact of the probability correction factor *c* in ref (22)
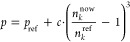
2*n*_k_^now^ represents the current number of molecules of species *k*, and *n*_*k*_^ref^ is the desired reference value. Essentially, this factor quantifies how the probability *p*_ref_ governing a specific molecular transfer must be adjusted in response to deviations from the equilibrium population. We systematically explored different values for correction factor *c*: Starting from the default value of 300 (see ref (22)), we also tried 200, 100, 50, and 10. Moreover, this factor was omitted completely to investigate whether we could achieve an equilibrium ratio between the ionic and neutral species. We also assessed how far this equilibrium deviated from the Markov chain model described in our previous study.^28^

### Polarizable Force Field

The force field parameters for the PIL were taken from ref (8). Initial Drude polarizable force field parameters of HPTSH^3–^ and HPTS^4–^ were generated from the Drude general force field (DGenFF)^29,30^ via FFParam,^31^ with additive force field parameters from CGenFF^32^ provided by CHARMM-GUI.^33−35^ QM geometry optimization was executed using Gaussian,^36^ employing the MP2 level of theory with the 6-31G* basis set. Calculations of dipole moment, molecular polarizability, and atomic charges were performed on the optimized geometry using the cc-pVDZ basis set, with CHELPG-derived partial charges^37^ accepted without scaling. As only a limited number of molecules containing heteroatoms are available in DGenFF, certain proposed parameters—particularly those involving bonds and angles with sulfur atoms—demonstrated a high penalty. Therefore, internal coordinates obtained from a short MD simulation were compared with QM coordinates. In instances of substantial discrepancy, QM and MD potential energy scans were conducted over the relevant bond or angle. The force field parameters were then iteratively adjusted to improve alignment, ensuring that alterations did not compromise the quality of the MD dipole moment and polarizability.

A script from Heid et al.^38,39^ was utilized to compute QM atomic polarizabilities, which entailed six single-point calculations on the QM-optimized geometry with Gaussian, using the Def2TZVP^40^ basis set. Each calculation involved the application of an electric field of 0.0008 au in one of the positive and negative *x*-, *y*-, and *z*-directions. Atomic dipoles were obtained from the wave functions with GDMA,^41^ and atomic polarizabilities were computed using the Heid et al. script.^38,39^ Hydrogen polarizabilities were aggregated with the heavy atoms to which they were bound. Given that gas phase QM polarizabilities generally exceed the desired values for MD simulations in solution,^42,43^ acquired atomic polarizabilities were scaled down using scaling factors proposed by Lemkul et al.^42^ In cases lacking specific recommendations from that reference, a universal factor of 0.85 was employed.

To eliminate the double-counting of London forces, the original nonpolarizable Lennard-Jones well depth ϵ_β_^*np*^ of each polarizable atom β was scaled^44^
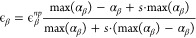
3

This incorporated atomic polarizabilities α_β_ and a scaling factor *s*, where max(α_β_) represents the highest atomic polarizability within the system. Scaling factors of 0.25 and 0.4 were examined for both PIL and HPTS^4–^/HPTSH^3–^.

Polarizable force field parameters of MeOH were procured from DGenFF^30^ and accepted without modification. The partial charges of MeOH_2_^+^ were established via QM, as previously outlined for HPTS^4–^/HPTSH^3–^. Parameters for internal coordinates were sourced from MeOH and supplemented with additional hydrogen. Neither MeOH nor MeOH_2_^+^ underwent Lennard-Jones scaling, as their force field parameters, sourced directly from the Drude CHARMM force field, were already optimized for MD simulations in solution. All force field parameters can be found in the ESI.

### Simulation Protocol

All simulation boxes were populated utilizing Packmol^45^ and equilibrated via a 5040 ps npT simulation. Consequent nVT production runs featuring a time step of 0.5 fs and a total duration of 50 ns were implemented with OpenMM.^46^ Both a velocity Verlet integrator and a Nosé–Hoover thermostat were incorporated into the simulation. Non-Drude particles were maintained at a temperature of 300 K, while Drude particles were held at 1 K. A “DrudeHardWall” parameter of 0.2 Å was employed to keep Drude particles in proximity to their parent atoms, with a Drude force constant of 1000 kcal/mol/Å^2^ and a Drude mass of 0.4 amu. The save frequency was set at 200 timesteps, and molecular charges were saved to monitor the protonation state of each molecule at every time step.

During the production phase, Protex executed proton transfers with initial transfer probabilities documented in Table 2, which were derived from one-dimensional QM scans.^28^ The transfer probabilities involving HPTSH^3–^ and MeOH _2_^+^ were set to unity to ensure the immediate transfer of hydrogens when a viable partner emerged. The protocol prohibited the transfer of protons back to HPTS^4–^ or MeOH, yet allowed transfers between MeOH_2_^+^ and MeOH.

**Table 2:** QM Probabilities to Transfer the Proton Once the Contact Distance *pxr* is Reached. The Probabilities Concerning the PIL Only Were Taken From ref (28)

donor	acceptor	probability *p*_ref_ (%)
HOAc	OAc^–^	68.4
HOAc	Im_1_	9.8
Im_1_H^+^	OAc^–^	99.4
Im_1_H^+^	Im_1_	20.1
HPTSH^3–^	OAc^–^	100.0
HPTSH^3–^	Im_1_	100.0
HPTSH^3–^	MeOH	100.0
MeOH_2_^+^	OAc^–^	100.0
MeOH_2_^+^	Im_1_	100.0
MeOH_2_^+^	MeOH	100.0

Transfer probabilities *p* of the pure IL were updated after each transfer to retain the equilibrium concentrations according to eq 2. To prevent the perpetual exchange of a single proton between the same pair of molecules, these molecules were rendered ineligible for subsequent transfers for the next ten update trials.

### Analysis of the Trajectories

Analysis of trajectories was carried out with the MDAnalysis^47,48^ package and self-written Python scripts. The exact protocols for analysis and additional measurements that had to be taken to handle some effects caused by proton transfers are described in detail in ref (22). In short, the saved charges were used to identify the momentary state (ionic or neutral) of each molecule. Thus, only time series where a molecule stayed in the same state for at least 25 ns were considered for calculating the diffusion coefficients. Additionally, these charges were used to calculate the collective translational dipole moment and, from that, the conductivity. In the case of the system with the photoacid, only the mean-square displacements between 750 and 1750 ps were used for the computations of the diffusion coefficients and the conductivity as only a few molecules keep their protonation states for much longer times. For the pure PIL, the range of 2-6 ns was analyzed to keep consistency with ref (8).

At each successful transfer event, the indices of the involved molecules were also saved. This information was used to follow the evolution of proton transfer chains that originated from HPTSH^3–^ in the system with the photoacid.

## Updates to Protex

Originally, Protex was developed specifically for pure PIL 1-methylimidazolium acetate. In this work, we present several new features of Protex to conduct simulations involving more species and allow for more precise handling of the proton transfer events. To accommodate molecules that did not exist at the beginning of the simulation, such as MeOH_2_^+^ or HPTS^4–^ in our case (see Table 1), an additional OpenMM simulation object was created. This new object contained a singular molecule of every possible species, enabling the initialization of templates for each acid/conjugate base pair.

### Equivalent Donors/Acceptors

**Figure 3:**
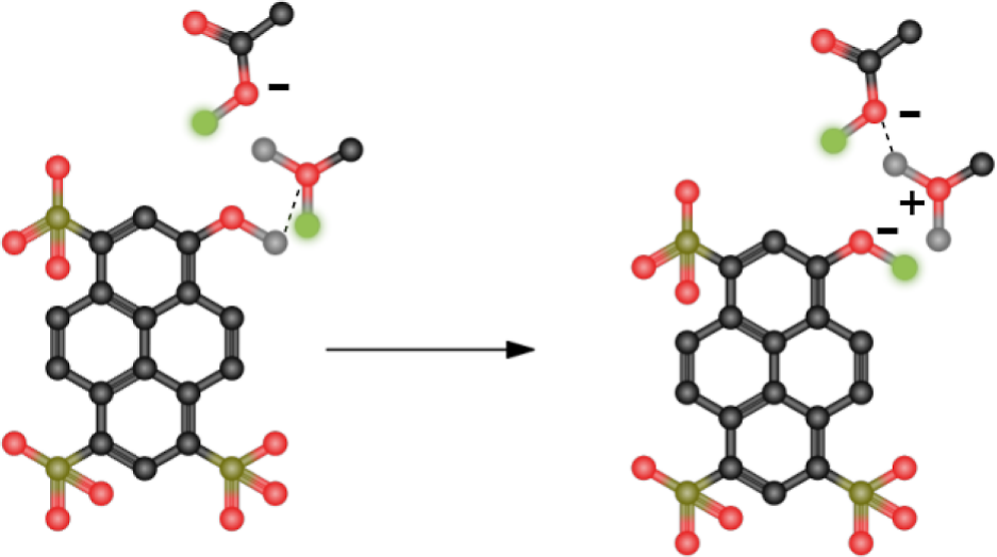
Disabled Grotthuss mechanism in the original Protex. After a proton transfer from HPTSH^3–^ to MeOH (left), there is now a very strong H-bond between the negatively charged oxygen and the transferred proton (right). The other acidic proton of MeOH_2_^+^ cannot be transferred to the nearby OAc^–^, as it has always been a real proton, even though the distance criterium (dashed line) is fulfilled. Dummy protons are shown in green.

The original program^8^ Protex revealed a shortcoming: Although both oxygen atoms of OAc^–^ are theoretically protonatable, only the one bearing the dummy hydrogen was actually susceptible to protonation due to the system’s current configuration. Similarly, despite both acidic protons of MeOH _2_^+^ being theoretically donatable, only the one that was initially a dummy hydrogen was capable of being deprotonated. This circumstance introduced a degree of unnatural behavior into the system, thereby impeding the Grotthuss mechanism, as depicted in Figure 3.

For instance, following a proton transfer from HPTSH^3–^ to MeOH, a pronounced attraction was observed between the newly formed MeOH_2_^+^‘s once-dummy hydrogen and the newly negatively charged oxygen of HPTS^4–^. This interaction prompted these two atoms to remain in close proximity, thereby inhibiting the transfer of the other acidic proton in MeOH_2_^+^ even in the presence of a nearby suitable acceptor. To address this limitation, a novel update method was devised in which equivalent atoms (i.e., the pre-existing hydrogen in MeOH of MeOH_2_^+^ and the oxygen atom of OAc^–^ without a dummy hydrogen) were also included in the consideration. In this approach, these equivalent atoms were used solely to compute distances between potential acceptors and donatable hydrogens, but the execution of the actual proton transfer remained unchanged from that of the previous version.

### Reorientation of the Acceptor

**Figure 4:**
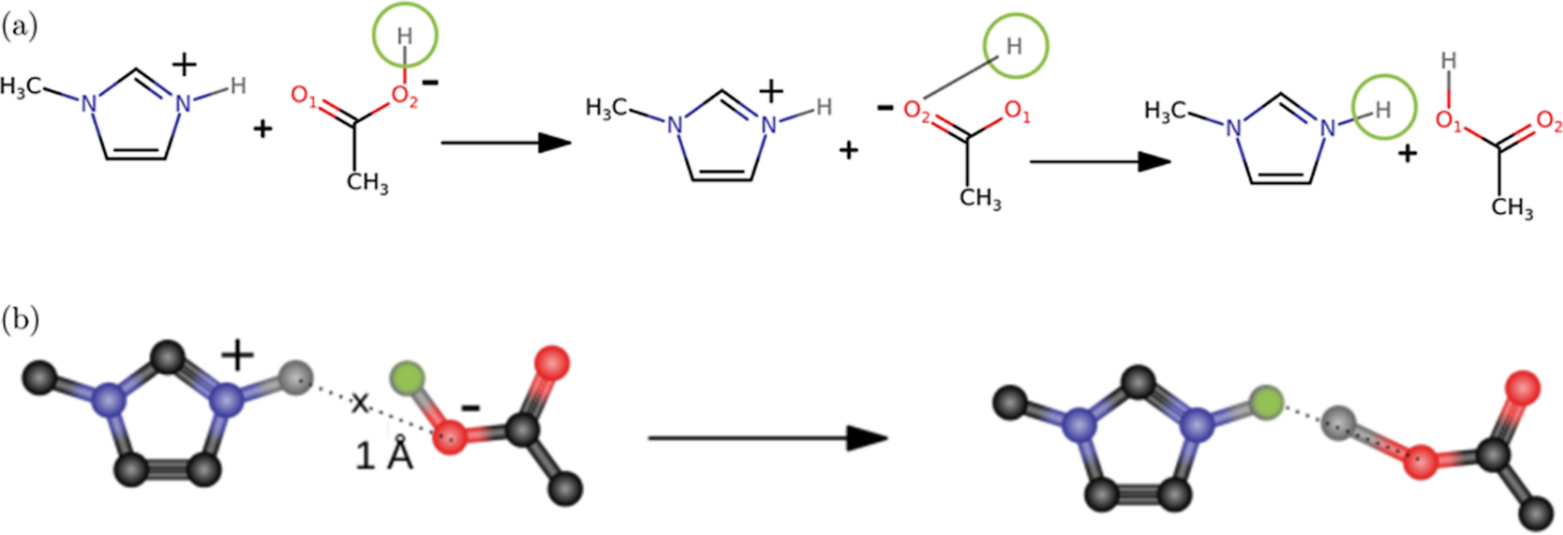
Reorientation of equivalent atoms (above) and determining the new position of the transferred H (below). (a) Exchanging the positions of the equivalent Os of OAc^–^. Dummy Hs are marked with a green circle. As the O without the dummy H(O1) is closer to the donated proton than O2, their positions are swapped. After the transfer, the position of the H of HOAc is also updated. The unnaturally long O–H bond in the middle step does not lead to problems, as there are no simulation steps during the reorientation. (b) The exact mechanism of setting the new position of the transferred H. The molecules before the transfer are shown on the left. The new position (marked with an *x*) is calculated to lie on the acceptor atom—donated H line, 1 Å from the acceptor. The right side shows the molecules after the transfer, with the H at its new position. Dummy atoms are shown in green.

The next iteration of the Protex program encompassed an advanced reorientation procedure for equivalent atoms, effectively ensuring a more accurate representation of the proton transfer process. This mechanism allowed for the exchange of positions between the two acidic protons in MeOH_2_^+^ or the two oxygen atoms in OAc^–^ before the transfer, thereby offering a more realistic depiction of the transferred hydrogen’s movement. In the instance of OAc^–^, adjustments were also made to the dummy hydrogen’s position to prevent undue extension of the O–H bond. This was accomplished by positioning the accepted hydrogen (H of HOAc) on the acceptor (O of OAc^–^)—donated H (H of Im_1_H^+^) line, at a distance of 1 Å from the acceptor atom, as illustrated in Figure 4. This resulted in the “new” bond length aligning closely with the equilibrium bond lengths of typical N–H and O–H bonds.

This procedure for resetting the position of the transferred hydrogen was also applied to transfers that did not involve OAc^–^, aiming to eliminate abrupt positional changes owing to the disappearance of one hydrogen and the concurrent appearance of another in the position originally occupied by the dummy hydrogen. This approach was adopted to generate more realistic diffusion and conductivity values. Although attempts were made to position the accepted hydrogen close to the donated hydrogen, this tactic resulted in unstable simulations due to excessively elongated new bonds.

### Blocked Transfers

We also fixed a minor error associated with blocking the donor–acceptor pair of a proton transfer for the subsequent ten update trials. Before this amendment, the pair remained blocked until ten successful transfers transpired. This protocol was modified to encompass the succeeding ten update trials, irrespective of the tally of successful transfers. We have implemented a procedure to save the list of blocked transfers at the end of each simulation run. This saved list can then be restored at the beginning of the next simulation, enabling us to restart simulations under conditions identical to those in the previous one ended.

### Distance-dependent Probabilities

**Figure 5:**
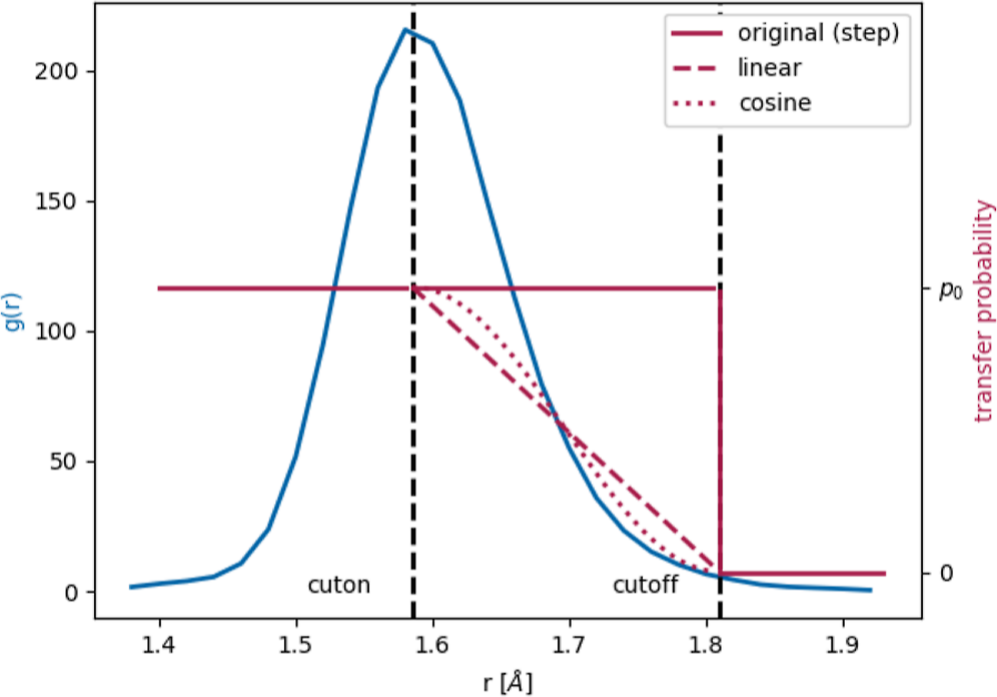
First coordination shell in the RDF (blue), as a basis for scaling down the transfer probability for larger distances. Black dashed lines show the cut-on and cutoff. The available functions are shown in red: no scaling (solid), linear (dashed), and cosine (dotted).

A new feature was introduced in Protex that facilitated scaling of the transfer probability as a function of the distance of the donor and acceptor atom. To effectively operationalize this feature, both a cut-on *r*_min_ distance and a cutoff *r*_max_ distance had to be explicitly delineated. The designated initial probability, *p*_0_, was applied to distances that were less than *r*_min_ and progressively reduced to zero for distances falling within the interval between *r*_min_ and *r*_max_. Both linear and cosine functions were devised for this scaling process, as depicted in Figure 5. The advantage of the cosine function is that the derivative is zero at *r*_min_ and *r*_max_. In the original Protex, only a step function at *r*_max_ was available.

**Figure 6:**
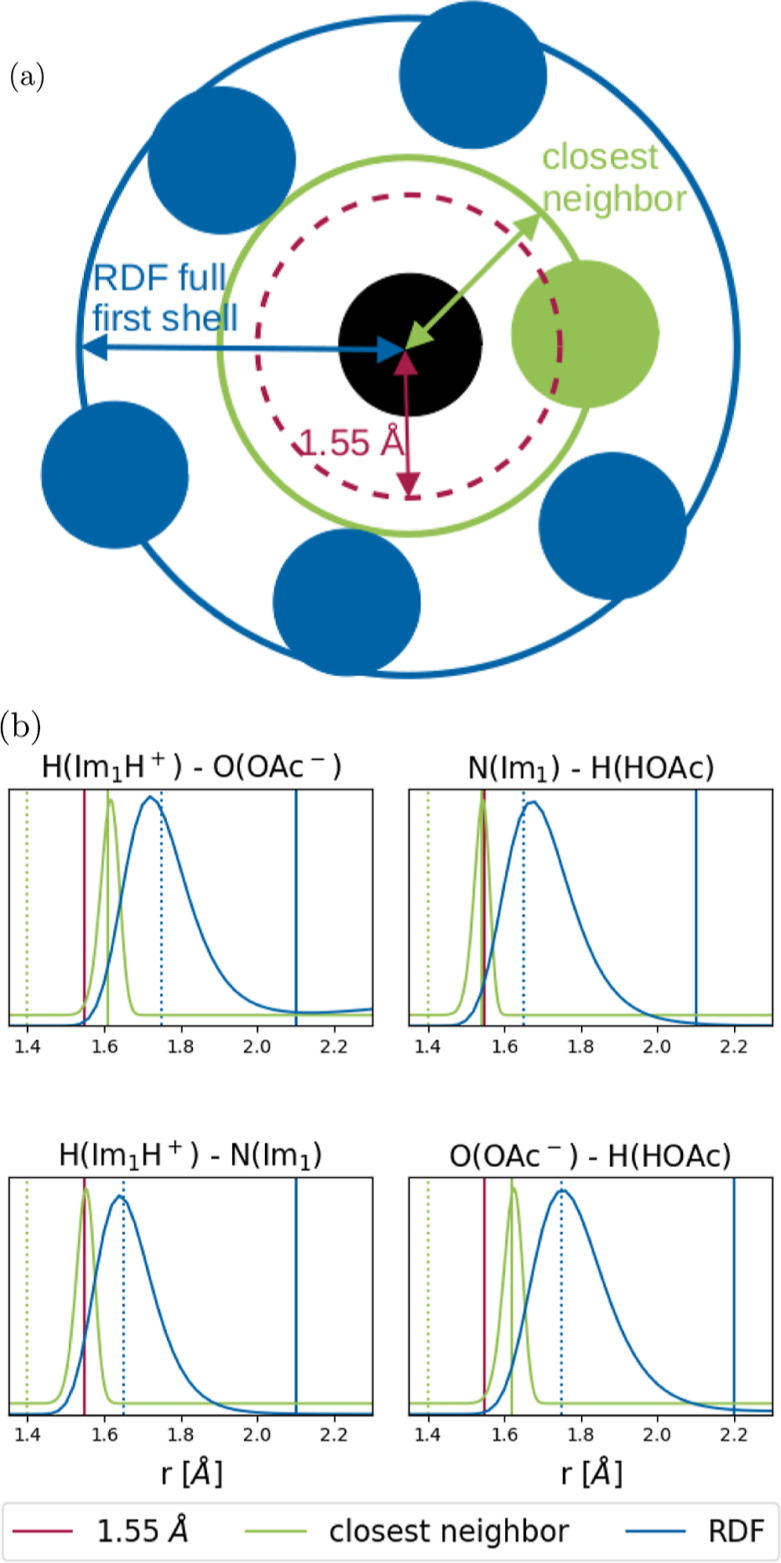
Illustration of the different methods for determining cutoff radii (above) and the corresponding functions (below). (a) The three possibilities for defining the cutoff radius around a central atom (black). Uniform cutoff at 1.55 Å (red), the whole first coordination shell (blue), and the closest neighbors method (green). (b) RDFs (blue) between deprotonatable hydrogens and possible acceptor atoms, and the closest distance between atoms of the concerned type (green) for each pair of species in the pure IL. The standard 1.55 Å cutoff is shown in red, the updated cut-on and cutoff values are shown in the corresponding color by a dashed and solid line, respectively.

To determine the most appropriate cut-on and cutoff distances, the shortest distance in each frame was calculated for each possible deprotonatable hydrogen and acceptor atom combination during a 50 ns nVT simulation without proton transfers. The radial distribution function (RDF) was also computed for each of these pairs. The first shell of the RDF includes all molecules that are in the first coordination shell of the central molecules, whereas there is only one closest neighbor for each molecule. It is important to note that we took the shortest distance to the closest neighbor over all pairs of the concerned type in each frame and not the average distance to the closest neighbor for each central molecule. These two functions, as well as their physical meaning, are shown in Figure 6. It can be seen that the standard 1.55 Å cutoff covers a varying proportion of molecules, depending on the species. This means that using a uniform cutoff for each pair, the probability of a successful transfer is falsified.

Special points of these two functions were used to test possible cut-on and cutoff distances individually for each pair of species, as shown in Figures 5 and 6. The full first shell of the RDF was defined by setting the cutoff around the maximum of the peak and the cutoff to the baseline after the peak. For the closest neighbors method, the range was shortened further, by choosing a cut-on below the onset of the peak, and a cutoff at its maximum. The corresponding values for the pure IL are summarized in Table 3.

**Table 3:** Cut-On and Cut-Off Distances for Each Reaction in the Pure IL, Using the Closest Neighbors and the Full First Shell in the RDF Methods

	closest neighbors		RDF first shell	
	cut-on [Å]	cutoff [Å]	cut-on [Å]	cutoff [Å]
Im_1_H^+^ + OAc^–^	1.40	1.61	1.75	2.10
Im_1_ + HOAc	1.40	1.54	1.65	2.10
Im_1_H^+^ + Im_1_	1.40	1.55	1.65	2.10
HOAc + OAc^–^	1.40	1.62	1.75	2.20

## Results and Discussion

### Reproducing the Markov chain

The Markov chain model outlined in ref (28) operates on the assumption that the number of contacts between species is solely determined by their concentrations. When a contact pair is identified, the decision regarding whether new species are formed or no reaction occurs depends on the reaction probability. Consequently, this process can lead to a decrease in the concentration of the initial compounds and an increase in the concentration of the final products. These updated concentrations are then used to identify new contact pairs that are eligible for the next proton transfer reactions.

However, in practical MD simulations, the number of contacts is not solely a function of species concentrations but also depends on their interactions. When two species exhibit strong attractive interactions, the likelihood of identifying contact pairs is higher than expected based on their concentrations. Conversely, if repulsive interactions are predominant, then there will be fewer contacts than expected given their concentrations.

The probability correction factor *c* in eq 2 is a remedy to counteract the influence of attractive and repulsive interactions and set up a system close to the prediction of the underlying Markov chain model. Nevertheless, it enforces an equilibrium situation that may not exist without the correction. Consequently, we tested the transient ratio between ionic and neutral species without the probability correction as well as with increasing strength of this correction factor.

#### Without Probability Correction

Our previous simulations on PILs^8,22^ started close to the final ratio of charged and neutral species. Consequently, an npT simulation without proton transfer at the given ratio is sufficient to determine the density of the system. In the subsequent nVT at this density, the proton transfers were switched on and led to fluctuations around the initial ratio. As these fluctuations were small, no significant density effects were expected, which justifies the application of an nVT instead of an npT simulation.

**Figure 7:**
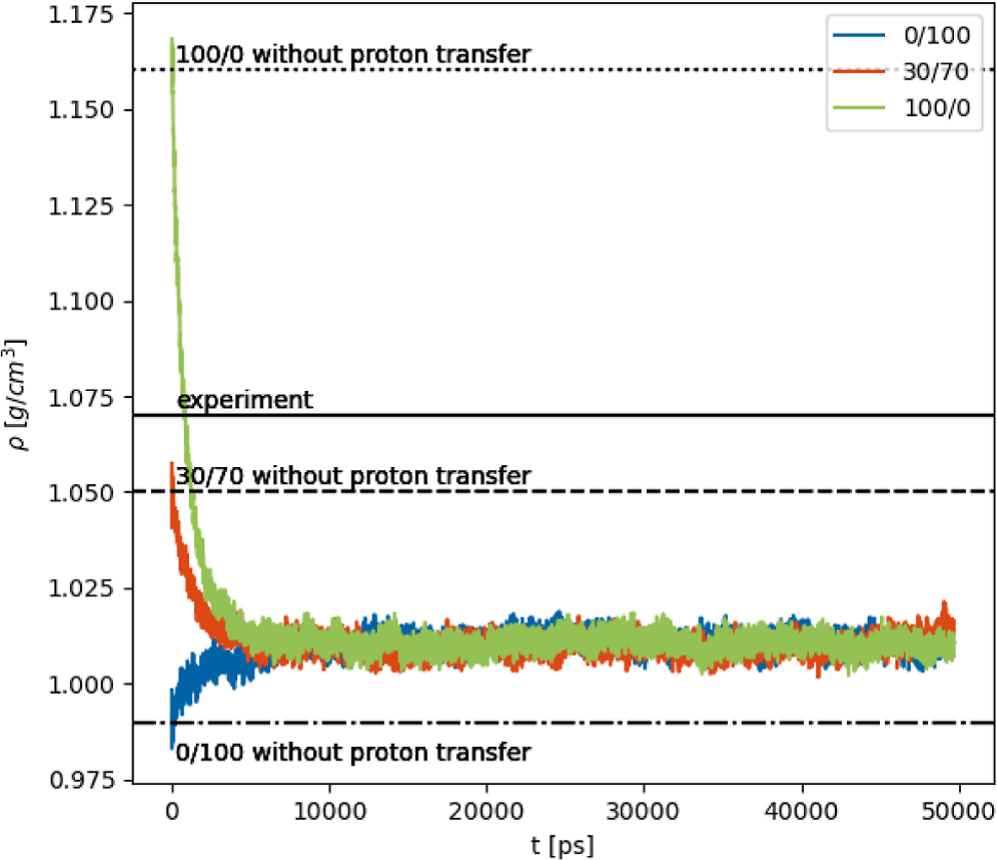
Time evolution of the density of the systems starting from 0% (blue), 30% (orange), and 100% (green) ionic species during simulations with proton transfers. Averages over three replicas each, with an additional rolling average with a window of 10 ps. The first data points were added afterward to show the quick change in density at the beginning of the simulation. The raw data of the individual replicas are shown in the ESI.

However, to test the robustness of our proton transfer setup, we now started at extreme conditions, 100% charged species (100/0 in Figure 7) and 0% charged species (0/100 in Figure 7). As these starting configurations are far away from the equilibrium, density effects are expected:^8^ due to the stronger Coulomb interactions between charged molecules, the density of the PIL increases with increasing mole fraction of the ions. To perform an unbiased simulation, we had to switch to npT simulations, despite the additional issues of the interactions of the barostat with the proton transfers. To keep consistency with the densities obtained in ref (8), bonds to hydrogens were fixed in these simulations as well. Furthermore, the transient box length also had to be tracked.

Interestingly, an equilibrium between charged and neutral species at a density of ρ = 1.01 g cm^–3^ is reached within 2 ns as shown in Figure 7, irrespective of the starting configuration (100/0, 30/70, or 0/100). In particular, the first proton transfer events resulted in large density changes. In (more or less) completely ionic systems, many 1-methylimidazolium acetate pairs eligible for proton transfer are detected. Given a proton transfer probability of 99.4% (see Table 2) for these pairs, many neutral species emerge rapidly, driving the density immediately to lower values. Starting from a neutral system, the density ρ also increases fast (blue line in Figure 7) despite the lower proton transfer probability of 9.8%. It seems that the vast excess of the neutral species is still sufficient to produce significant amounts of Im_1_H^+^ and OAc^–^. Nevertheless, the fast initial increase is accompanied by a slower process to reach the final equilibrium density.

However, the equilibrium density ρ = 1.01 g cm^–3^ in these npT simulations neither agrees with the value of a 30/70 simulation without proton transfer nor with the experimental value of 1.07 g cm^–3^.^49,50^ In fact, even starting at 30/70, the npT proton transfer simulation (orange line in Figure 7) results in a density of ρ = 1.01 g cm^–3^ again. At this density, a ratio of around 10% charged to 90% neutral species is observed,^8^ which contradicts the Markov model^28^ at first sight but can be explained by the particular mutual interaction of the species. In principle, the concentrations in the Markov model should be adjusted by a radial distribution factor describing the accumulation or depletion of a species around another species due to attractive and repulsive forces.

#### Influence of the Probability Correction

In our recent study,^8^ the determination of a ratio involving 30% charged and 70% neutral species was accomplished through an analysis of key parameters, including density, diffusion coefficients, and the dielectric spectrum of the PIL. For example, the computational density values for the 30/70 system are close to the experimental density, as depicted in Figure 7. One plausible approach to achieve the desired density for the npT simulation is to adjust the probabilities outlined in Table 2. However, this adjustment process may necessitate multiple iterations of trial-and-error runs, as all possible proton transfer reactions are coupled and determine the final equilibrium. Furthermore, discrepancies may arise between the newly determined probabilities and those obtained from rigorous QM scans.

**Figure 8:**
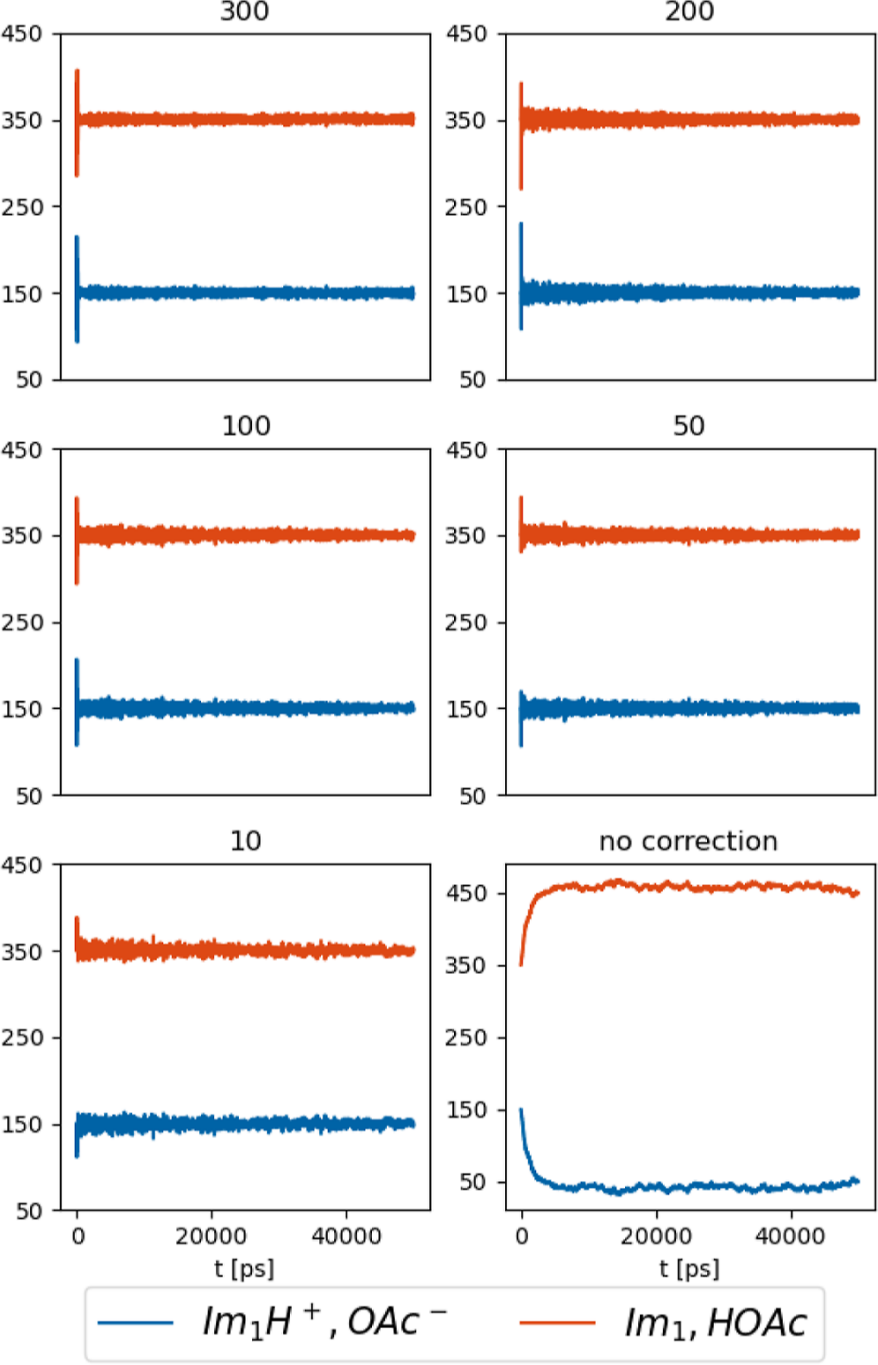
Time evolution of the number of molecules for each species with varied probability correction factors *c*. Averaged data for six (*c* = 300) or three (*c* = 200, 100, 50, 10, no correction) replicas. The individual replicas are shown in the ESI.

A more streamlined alternative entails the utilization of eq 2. Within this framework, some probabilities governing particular proton transfer events are systematically enhanced if the corresponding product concentration falls below the desired threshold. Conversely, these probabilities can be reduced if the reactant concentrations are below expectations. As evident from Figure 8, the application of probability corrections effectively maintains the population of each species in the proximity of the targeted equilibrium values. It is noteworthy that a correction factor *c* with a value of 10 proves sufficient to reach the desired concentrations, with more potent corrections yielding no discernible enhancements and thus being deemed unnecessary.

### Better Description of the Grotthuss Mechanism

**Figure 9:**
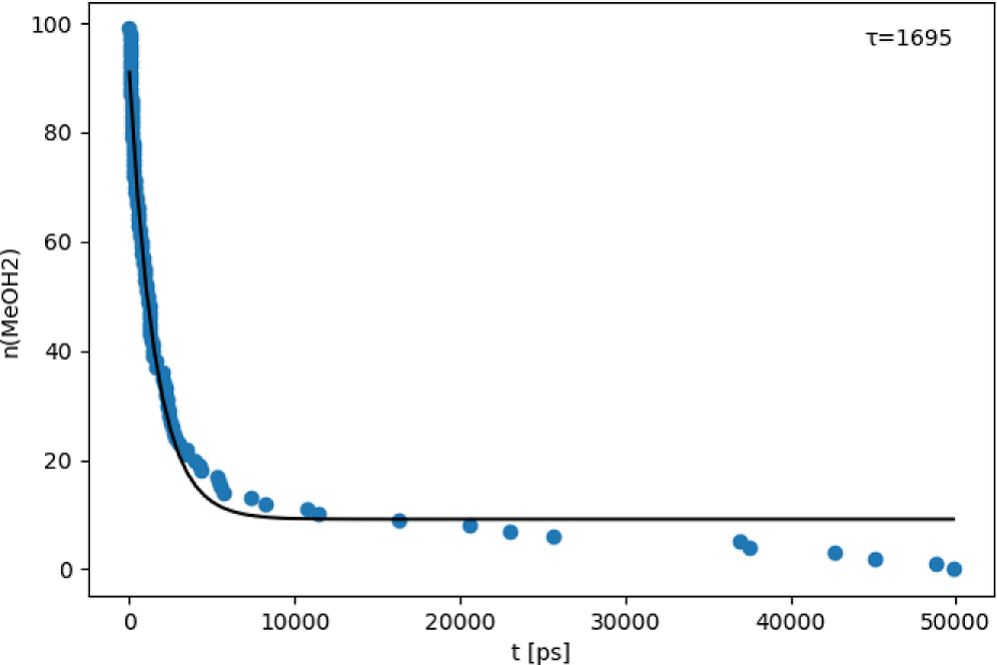
Time evolution of the number of protonated methanol molecules (blue) and exponential fit (black). *t* = 0 denotes the time where the corresponding MeOH_2_^+^ was formed from a MeOH by accepting a proton from HPTSH^3–^. Pooled data from six replicas with 18 HPTSH^3–^ each.

The updates to Protex had a significant effect on the modeling of the Grotthuss mechanism. As mentioned above, the original version did not allow the deprotonation of both Hs of MeOH_2_^+^, whereas cases of proton hopping over a chain of MeOH_2_^+^ ions were observed with the reorient update. This also had a great effect on the transfer of the excess proton, as can be seen in Table 4, which shows the number of proton transfers in the chain of transfers started by the deprotonation of the photoacid. Using the original approach, the proton of HPTSH^3–^ was first transferred to solvent methanol. In most cases, this led to a strong attraction between the newly transferred H of MeOH_2_^+^ and the O of HPTSH^4–^, as already depicted in Figure 3. Since transferring the other acidic H of MeOH_2_^+^ was forbidden, the chain of proton transfers was broken after the first transfer event. This was no longer the case after the updates. With this improvement, an exponential decay in the concentration of protonated methanol was observed, as shown in Figure 9. A similar behavior was also observed by our experimental partners, which will be discussed in an upcoming publication.

**Table 4:** Average Length of Proton Transfer Chains Starting From HPTSH^3–^ as a Function of the Cut-Off Radius (*pxr*) and the Interval Between Transfers (*pxi*), Using the Original Approach (Top) and the Reorient Method (Middle). Average Decay Constants (in ps) for the Consumption of MeOH _2_^+^ as a Function of the Cut-Off Radius (*pxr*) and the Interval Between Transfers (*pxi*), Using the Reorient Method (Bottom)

average proton transfer chain length (original)				
	pxr [Å]			
pxi [ps]	1.55	1.58	1.60	1.62
0.5	1	1	1	1
1.0	1	1	1	2
3.0	1	2	1	1
5.0	1	1	1	3

average proton transfer chain length (reorient)				
	pxr [Å]			
pxi [ps]	1.55	1.58	1.60	1.62
0.5	8	19	13	14
1.0	16	10	11	11
3.0	11	12	16	14
5.0	6	15	28	15

decay constants [ps] (reorient)				
	*pxr* [Å]			
pxi [ps]	1.55	1.58	1.60	1.62
0.5	209	137	45	13
1.0	576	9	107	136
3.0	1142	120	359	98
5.0	1847	323	388	138

As expected, increasing the cutoff radius *pxr* and shortening the exchange interval *pxi* led to more transfers during the simulation. A larger cutoff radius includes more pairs that meet the distance criteria, while a shorter exchange interval results in more frequent proton transfers. This increased transfer rate is reflected in the decay constants observed in the consumption of protonated methanol, as shown in Table 4. A higher transfer rate accelerates this process, allowing us to fine-tune our parameters to better match experimental results. However, we recommend using an exchange interval of 7–10 ps, in accordance with the collision frequency determined by Jacobi et al.^28^ The cutoff radius should be individually defined for each pair of species, as discussed above.

### Effect of the Proton Exchange Radius on the Transport Properties

#### Conductivity

**Figure 10:**
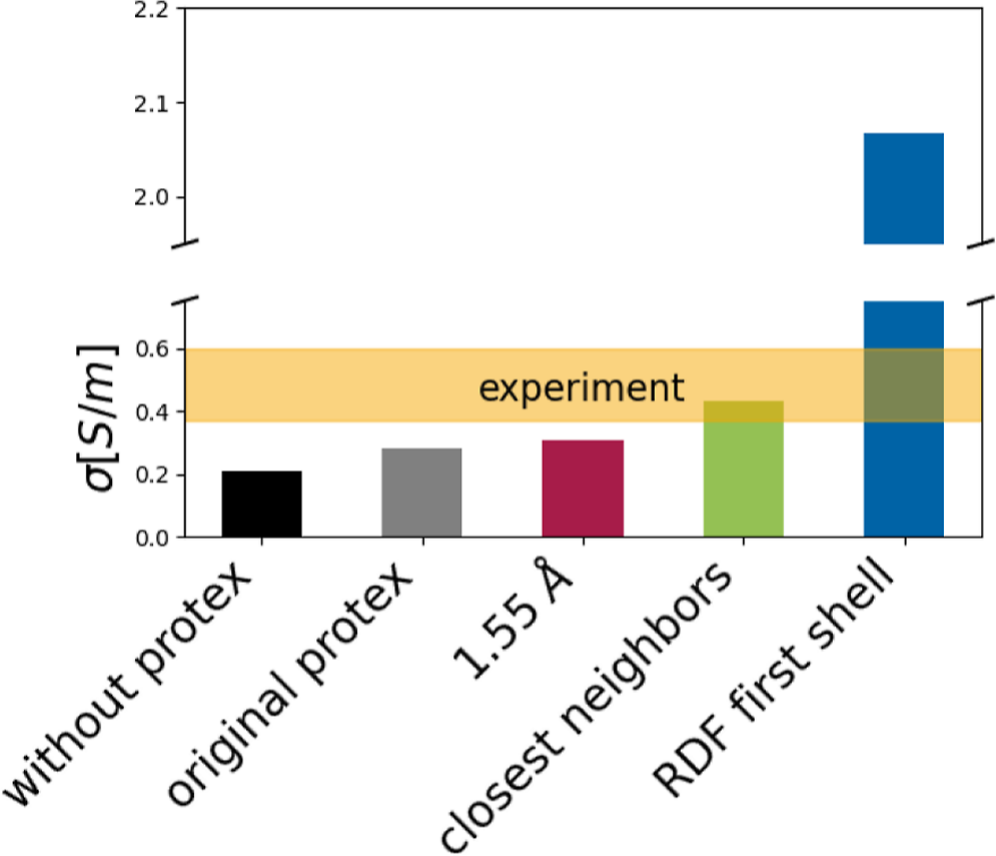
Conductivity σ of the pure IL calculated without Protex (black), with the original version (gray) and the updated Protex (the colors correspond to Figure 6: red: uniform 1.55 Å cutoff; green: individual cut-offs based on the shortest distance between the concerned atom types, blue: individual cut-offs based on the whole first coordination shell in the RDF). The range of reported experimental conductivities^49,51^ is shown by the yellow bar.

As can be seen in Figure 10, the conductivity of the pure IL simulated without proton transfers (black box) is significantly lower than the experimental data. Including proton transfers increased the conductivity (gray box), in accordance with expectations and findings from Joerg and Schröder.^8^ The reorient method (red box) also led to a slight increase in conductivity due to the larger number of possible proton transfers involving OAc^–^. Increasing the cutoff from the standard, uniform 1.55 Å to individual values based on the average closest distance between atoms of the concerned types (green box) led to an even better agreement with experimental findings. A further increase in the cutoff, e.g., to cover most of the first coordination shell, is no longer beneficial, as it leads to an exaggerated conductivity due to the greater distances the transferred protons are forced to “hop” over during the transfer.

#### Diffusion

**Figure 11:**
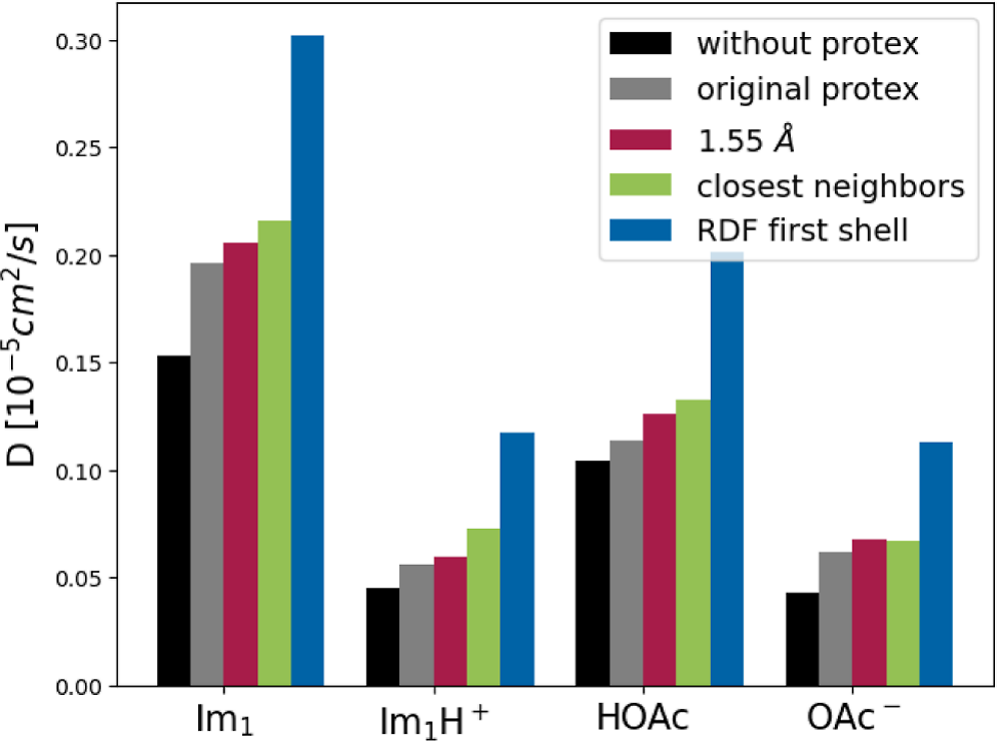
Diffusion coefficients *D* of each species in the pure IL calculated without Protex (black), with the original version (gray) and the updated Protex (red: uniform, low cutoff; green: individual cut-offs based on the shortest distance between the concerned atom types, blue: individual cut-offs based on the RDF).

The calculated diffusion coefficients of the pure IL in Figure 11 are in good agreement with the previous study.^8,22^

The slight increase in diffusion with Protex that was previously observed is also reproduced here. This increase is especially pronounced in the case of imidazole, which the cancellation of cage effects can explain.^22^ The difference between various Protex versions is negligible compared to the tremendous increase when the complete first coordination shell is used.

The proton jumps over larger distances are major reason that the conductivity increase is more pronounced than that of the diffusion. Nevertheless, the diffusion also profits significantly from the increased number of transfers due to the larger *pxr*. Exchanging a proton, an ion pair becomes two neutral species, which does not increase the mobility of charge carriers per se but destroys ion cages.^22,52^ Weaker ion cages enhance the mobility of individual ions. However, the newborn neutral 1-methylimidazole is still covered by several anions of the former ion cage, which puts it in an unfavorable position.^22^ The repulsion of the anions may kick the central 1-methylimidazole thereby increasing its diffusion.

Altogether, the setup of the proton exchange radius becomes a crucial force field parameter.

## Conclusions

The workflow and mechanisms of Protex were updated to be able to handle more complex systems than what it was initially developed for, which is an essential step on the way to generalizing Protex for arbitrary systems. A way was found to handle two chemically equivalent atoms in the same molecule provided that their positions can be swapped without distorting the structure of the molecule. This enabled simulation of the Grotthus mechanism, cases of which were also observed.

The effects of varying the cutoff radius and exchange interval were investigated, and a protocol was developed to define better cutoff radii individually for each possible pair of species. It was shown that choosing the cutoff radius individually for each pair of species is crucial for reproducing experimental transport properties. Scaling the transfer probability based on the distance was also implemented.

In the future, it is planned to include dummy protons on all protonatable acceptor atoms and to make all acidic hydrogens capable of being turned into dummy protons. This way, Protex will be better suited for other systems as well, and less setup will be needed from the user. Cases where swapping the positions of equivalent atoms is not as easily possible as in acetate or protonated methanol are also covered. Additionally, with this method, manually repositioning atoms will no longer be necessary, which will hopefully further increase the stability of the simulations.

## Data Availability

Protex is available on GitHub (https://github.com/cbc-univie/protex) free of charge. Test systems that can be used to set up a simulation with Protex are also included.

## Supporting Information Available

•ESI.pdf: Force field parameters for HPTSH^3–^/HPTS^4–^ and MeOH/MeOH_2_^+^; exemplary input file highlighting the updates; conductivity, diffusion, and density data used for the plots in the article (PDF)
