# Supplementary material for: Updates to Protex for Simulating Proton Transfers in an Ionic Liquid
Source: J Phys Chem B. 2024 Apr 1;128(14):3416–26. doi: 10.1021/acs.jpcb.3c07356 (PMC11017242; doi:10.1021/acs.jpcb.3c07356)
Supplement: Supplementary file 1 — jp3c07356_si_001.pdf [file jp3c07356_si_001.pdf]

Supporting Information for Updates to Protex for Simulating Proton Transfers in an Ionic Liquid

Márta Gődény,[†,‡] Florian Joerg,[†,‡] Maximilian P.-P. Kovar,[†] and Christian Schröder[*,†]

†*University of Vienna, Faculty of Chemistry, Department of Computational Biological Chemistry, Währinger Straße 17, 1090 Vienna, Austria*

‡*University of Vienna, Vienna Doctoral School in Chemistry (DoSChem), Währinger Straße 42, 1090 Vienna, Austria*

E-mail: christian.schroeder@univie.ac.at

Parametrization

The original atomic parameters suggested by FFParam and the optimized parameters of $HPTS^{4-}$ and $HPTSH^{3-}$ are shown in tables 1 and 2. A structure with the atom labels corresponding to these tables is shown in fig. 1. The originally suggested and optimized internal coordinates are shown in tables 3-5. Mainly, bonds and angles containing sulfur atoms needed to be optimized. Many of the other parameters already resulted in a good agreement with QM potentials.

The force field parameters of MeOH were accepted from DGenFF without change. The parameters for $MeOH_2^+$ were derived using these as a starting point. The atomic parameters are shown in tables 6 and 7. The internal coordinates are shown in tables 8-10. These were taken directly from DGenFF, and the DUMH atom type was added.

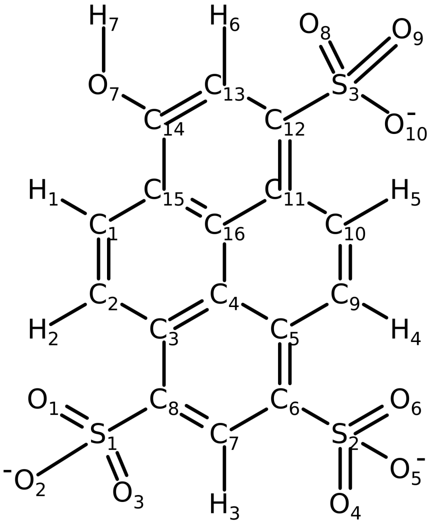

Figure 1: Atom labels of HPTSH.$^{3-}$ The atoms of $HPTS^{4-}$ were labeled analogously after the dummy H (H7) was added.

Table 1: **Atomic parameters of $HPTS^{4-}$, as first suggested by FFParam and after optimization. The charge of oxygens was assigned to their respective lone pairs and the polarizabilities of hydrogens to their neighboring heavy atoms. Thole parameters were not optimized.**

atom	atom type	charge [e]		α [Å^3]		Thole [Å]
		DGenFF	optimized	DGenFF	optimized	
C1	CD2R6A	-0.115	-0.159	-1.5075	-1.8600	1.2701
C2	CD2R6A	-0.115	-0.155	-1.5075	-1.8900	1.2701
C3	CD2R6A	0.011	0.060	-1.5075	-1.9300	1.2701
C4	CD2R6A	-0.020	0.047	-1.5075	-2.0200	1.2701
C5	CD2R6A	0.011	0.063	-1.5075	-1.9200	1.2701
C6	CD2R6A	-0.406	-0.166	-1.5075	-2.3300	1.2701
C7	CD2R6A	-0.293	-0.109	-1.5075	-1.4800	1.2701
C8	CD2R6A	-0.406	-0.160	-1.5075	-2.3200	1.2701
S1	SD30A	1.349	1.150	-1.0000	-1.6000	1.3000
O1	OD2C2B	0.000	0.000	-0.9310	-0.8967	1.0830
O2	OD2C2B	0.000	0.000	-0.9310	-0.8967	1.0830
O3	OD2C2B	0.000	0.000	-0.9310	-0.8967	1.0830
S2	SD30A	1.349	1.135	-1.0000	-1.5900	1.3000
O4	OD2C2B	0.000	0.000	-0.9310	-0.8967	1.0830
O5	OD2C2B	0.000	0.000	-0.9310	-0.8967	1.0830
O6	OD2C2B	0.000	0.000	-0.9310	-0.8967	1.0830
C9	CD2R6A	-0.115	-0.166	-1.5075	-1.9000	1.2701
C10	CD2R6A	-0.115	-0.182	-1.5075	-1.7100	1.2701
C11	CD2R6A	0.011	0.081	-1.5075	-2.0800	1.2701
C12	CD2R6A	-0.406	-0.187	-1.5075	-2.1800	1.2701
C13	CD2R6A	-0.683	-0.238	-1.5075	-1.8000	1.2701
C14	CD2R6A	0.395	0.341	-1.5075	-1.8700	1.2701
C15	CD2R6A	-0.309	-0.042	-1.5075	-2.1100	1.2701
C16	CD2R6A	-0.020	-0.090	-1.5075	-1.9600	1.2701
O7	OD30E	0.000	0.000	-1.0000	-1.1800	1.3000
S3	SD30A	1.349	1.185	-1.0000	-1.5900	1.3000
O8	OD2C2B	0.000	0.000	-0.9310	-0.9000	1.0830
O9	OD2C2B	0.000	0.000	-0.9310	-0.9000	1.0830
O10	OD2C2B	0.000	0.000	-0.9310	-0.9000	1.0830
H1	HDR6A	0.115	0.044			
H2	HDR6A	0.115	0.091			
H3	HDR6A	0.305	0.090			
H4	HDR6A	0.115	0.099			
H5	HDR6A	0.115	0.098			
H6	HDR6A	0.375	0.076			
H7	DUMH		0.000			
LPO11	LPDO1	-0.325	-0.351			
LPO12	LPDO1	-0.325	-0.351			
LPO21	LPDO1	-0.325	-0.336			
LPO22	LPDO1	-0.325	-0.336			
LPO31	LPDO1	-0.325	-0.350			
LPO32	LPDO1	-0.325	-0.350			
LPO41	LPDO1	-0.325	-0.335			
LPO42	LPDO1	-0.325	-0.335			
LPO51	LPDO1	-0.325	-0.344			
LPO52	LPDO1	-0.325	-0.344			
LPO61	LPDO1	-0.325	-0.344			
LPO62	LPDO1	-0.325	-0.344			
LPO71	LPDO1	-0.381	-0.337			
LPO72	LPDO1	-0.381	-0.337			
LPO81	LPDO1	-0.325	-0.352			
LPO82	LPDO1	-0.325	-0.352			
LPO91	LPDO1	-0.325	-0.352			
LPO92	LPDO1	-0.325	-0.352			
LPO101	LPDO1	-0.325	-0.352			
LPO102	LPDO1	-0.325	-0.352			

Table 2: Atomic parameters of $HPTSH^{3-}$, as first suggested by FFParam and after optimization.

atom	atom type	charge [e]		α [$Å^3$]		Thole [Å]
		DGenFF	optimized	DGenFF	optimized	
C1	CD2R6A	-0.114	-0.151	-1.5075	-1.4800	1.2701
C2	CD2R6A	-0.115	-0.170	-1.5075	-1.3500	1.2701
C3	CD2R6A	0.011	0.148	-1.5075	-1.3400	1.2701
C4	CD2R6A	-0.020	-0.039	-1.5075	-1.4300	1.2701
C5	CD2R6A	0.011	0.094	-1.5075	-1.4700	1.2701
C6	CD2R6A	-0.406	-0.150	-1.5075	-1.7700	1.2701
C7	CD2R6A	-0.293	-0.028	-1.5075	-1.2100	1.2701
C8	CD2R6A	-0.406	-0.179	-1.5075	-1.5600	1.2701
S1	SD30A	1.349	1.129	-1.0000	-1.1000	1.3000
O1	OD2C2B	0.000	0.000	-0.9310	-0.7367	1.0830
O2	OD2C2B	0.000	0.000	-0.9310	-0.7367	1.0830
O3	OD2C2B	0.000	0.000	-0.9310	-0.7367	1.0830
S2	SD30A	1.349	1.107	-1.0000	-1.4200	1.3000
O4	OD2C2B	0.000	0.000	-0.9310	-0.7400	1.0830
O5	OD2C2B	0.000	0.000	-0.9310	-0.7400	1.0830
O6	OD2C2B	0.000	0.000	-0.9310	-0.7400	1.0830
C9	CD2R6A	-0.115	-0.128	-1.5075	-1.2700	1.2701
C10	CD2R6A	-0.115	-0.182	-1.5075	-1.3600	1.2701
C11	CD2R6A	0.011	0.125	-1.5075	-1.3300	1.2701
C12	CD2R6A	-0.405	-0.153	-1.5075	-1.5000	1.2701
C13	CD2R6A	-0.197	-0.225	-1.5075	-1.3000	1.2701
C14	CD2R6A	0.103	0.177	-1.5075	-1.2200	1.2701
C15	CD2R6A	0.014	0.037	-1.5075	-1.3900	1.2701
C16	CD2R6A	-0.019	-0.059	-1.5075	-1.3900	1.2701
O7	OD31A	0.000	0.000	-0.8315	-0.9700	0.7494
S3	SD30A	1.349	1.159	-1.0000	-1.1100	1.3000
O8	OD2C2B	0.000	0.000	-0.9310	-0.7433	1.0830
O9	OD2C2B	0.000	0.000	-0.9310	-0.7433	1.0830
O10	OD2C2B	0.000	0.000	-0.9310	-0.7433	1.0830
H1	HDR6A	0.115	0.083			
H2	HDR6A	0.115	0.122			
H3	HDR6A	0.305	0.084			
H4	HDR6A	0.115	0.120			
H5	HDR6A	0.115	0.118			
H6	HDR6A	0.210	0.100			
H7	HDP1A	0.420	0.371			
LPO11	LPDO1	-0.325	-0.340			
LPO12	LPDO1	-0.325	-0.340			
LPO21	LPDO1	-0.325	-0.317			
LPO22	LPDO1	-0.325	-0.317			
LPO31	LPDO1	-0.325	-0.339			
LPO32	LPDO1	-0.325	-0.339			
LPO41	LPDO1	-0.325	-0.317			
LPO42	LPDO1	-0.325	-0.317			
LPO51	LPDO1	-0.325	-0.331			
LPO52	LPDO1	-0.325	-0.331			
LPO61	LPDO1	-0.325	-0.332			
LPO62	LPDO1	-0.325	-0.332			
LPO71	LPD	-0.269	-0.277			
LPO72	LPD	-0.268	-0.277			
LPO81	LPDO1	-0.325	-0.338			
LPO82	LPDO1	-0.325	-0.338			
LPO91	LPDO1	-0.325	-0.332			
LPO92	LPDO1	-0.325	-0.332			
LPO101	LPDO1	-0.325	-0.332			
LPO102	LPDO1	-0.325	-0.332			

Table 3: Bond parameters of $HPTS^{4-}/HPTSH^{3-}$, as suggested by FFParam and after optimization (the original parameters were accepted in cases where no optimized value is shown).

atom type	atom type	k [kcal mol^{-1} Å^{-2}]		r_{eq} [Å]	
		DGenFF	optimized	DGenFF	optimized
CD2R6A	SD30A	200.000		1.810	
CD2R6A	HDR6A	340.00		1.080	
SD30A	OD2C2B	155.500	320.500	2.036	1.500
CD2R6A	OD31A	334.300		1.411	
CD2R6A	OD30E	334.300	350.300	1.411	1.311
OD31A	HDP1A	536.500		0.970	
OD30E	DUMH	536.500		0.970	
LPDO1	OD2C2B	0.000		0.000	
LPDO1	OD30E	0.000		0.000	

Table 4: Angle parameters of $HPTS^{4-}/HPTSH^{3-}$, as suggested by FFParam and after optimization (the original parameters were accepted in cases where no optimized value is shown).

atom type	atom type	atom type	k [kcal mol^{-1} rad^{-2}]		θ_{eq} [°]	
			DGenFF	optimized	DGenFF	optimized
CD2R6A	CD2R6A	CD2R6A	40.000		120.000	
CD2R6A	CD2R6A	HDR6A	30.000		120.000	
CD2R6A	CD2R6A	SD30A	43.000	20.000	113.200	118.200
CD2R6A	SD30A	OD2C2B	60.000		104.250	
OD2C2B	SD30A	OD2C2B	60.000	105.000	116.000	112.800
CD2R6A	CD2R6A	OD30E	45.200	90.200	120.000	123.000
CD2R6A	CD2R6A	OD31A	75.200		122.000	
CD2R6A	OD31A	HDP1A	65.000		108.000	
CD2R6A	OD30E	DUMH	65.000		108.000	

Table 5: Dihedral parameters of $HPTS^{4-}/HPTSH^{3-}$, as suggested by FFParam (dihedral parameters were not optimized).

atom type	atom type	atom type	atom type	k [kcal mol^{-1}]	n	δ [°]
CD2R6A	CD2R6A	CD2R6A	CD2R6A	2.800	2	180.00
CD2R6A	CD2R6A	CD2R6A	HDR6A	4.200	2	180.00
HDR6A	CD2R6A	CD2R6A	HDR6A	2.400	2	180.00
HDP1A	OD31A	CD2R6A	CD2R6A	0.990	2	180.00
DUMH	OD30E	CD2R6A	CD2R6A	0.990	2	180.00
OD31A	CD2R6A	CD2R6A	CD2R6A	3.100	2	180.00
CD2R6A	CD2R6A	CD2R6A	OD30E	3.100	2	180.000
OD31A	CD2R6A	CD2R6A	HDR6A	4.200	2	180.00
OD30E	CD2R6A	CD2R6A	HDR6A	4.200	2	180.000
CD2R6A	CD2R6A	CD2R6A	SD30A	1.580	1	180.000
CD2R6A	CD2R6A	CD2R6A	SD30A	1.450	2	180.000
CD2R6A	CD2R6A	SD30A	OD2C2B	0.200	3	0.000
SD30A	CD2R6A	CD2R6A	HDR6A	0.050	2	0.000
SD30A	CD2R6A	CD2R6A	HDR6A	0.270	1	0.000

Table 6: Atomic parameters of MeOH.

atom	atom type	charge [e]	α [Å^3]	Thole [Å]
C1	CD33B	-0.140	-1.000	1.3
O1	OD31H	0.000	-1.028	1.3
HO1	HDP1A	0.360		
HO2	DUMH	0.000		
H1A	HDA3B	0.080		
H1B	HDA3B	0.080		
H1C	HDA3B	0.080		
LP1A	LPD	-0.230		
LP1B	LPD	-0.230		

Table 7: Atomic parameters of $MeOH_2^+$.

atom	atom type	charge [e]	α [Å^3]	Thole [Å]
C1	CD33B	-0.138	-1.000	1.3
O1	OD31H	0.000	-1.028	1.3
HO1	HDP1A	0.481		
HO2	HDP1A	0.481		
H1A	HDA3B	0.194		
H1B	HDA3B	0.194		
H1C	HDA3B	0.194		
LP1A	LPD	-0.203		
LP1B	LPD	-0.203		

Table 8: Bond parameters for MeOH/$MeOH_2^+$.

atom type	atom type	k [kcal mol^{-1} Å^{-2}]	r_{eq} [Å]
OD31H	CD33B	430.00	1.425
OD31H	HDP1A	536.50	0.970
OD31H	DUMH	536.50	0.970
CD33B	HDA3B	322.00	1.111
OD31H	LPD	0.00	0.000

Table 9: Angle parameters of MeOH/$MeOH_2^+$.

atom type	atom type	atom type	k [kcal mol^{-1} rad^{-2}]	θ_{eq} [°]	k_{UB} [kcal mol^{-1} rad^{-2}]	S_0 [Å]
HDA3B	CD33B	HDA3B	35.50	108.400	5.400	1.802
HDP1A	OD31H	HDP1A	35.50	120.000	5.400	1.802
DUMH	OD31H	HDP1A	35.50	120.000	5.400	1.802
CD33B	OD31H	HDP1A	58.20	106.000	0.000	0.000
CD33B	OD31H	DUMH	58.20	106.000	0.000	0.000
OD31H	CD33B	HDA3B	61.00	108.890	0.000	0.000

Table 10: Dihedral parameters of MeOH/$MeOH_2^+$.

atom type	atom type	atom type	atom type	k [kcal mol^{-1}]	n	δ [°]
HDP1A	OD31H	CD33B	HDA3B	0.179	3	0.00
DUMH	OD31H	CD33B	HDA3B	0.179	3	0.00

Updated input file

An input file for starting a simulation with `protex` is given below to illustrate the updates we made (shown in blue).

```
import sys
import warnings
import os
from pathlib import Path

from simtk.openmm.app import StateDataReporter, PDBReporter, DCDReporter
# barostat for npT
from simtk.openmm import XmlSerializer, MonteCarloBarostat
from simtk.openmm.app import CharmmCrdFile, CharmmParameterSet,
    CharmmPsfFile
from simtk.openmm.app import PME, HBonds
from simtk.openmm.app import Simulation
from simtk.unit import angstroms, kelvin, picoseconds, atmosphere
from simtk.openmm import (
        OpenMMException,
        Platform,
        Context,
        DrudeLangevinIntegrator,
        DrudeNoseHooverIntegrator,
        XmlSerializer,
    )

import protex
from protex.testsystems import generate_im1h_oac_dummy_system, OAC_HOAC,
    IM1H_IM1
from protex.system import ProtexSystem, ProtexTemplates
from protex.reporter import ChargeReporter, DrudeTemperatureReporter,
    EnergyReporter
```

```python
# new update class
from protex.update import KeepHUpdate, StateUpdate

allowed_updates = {}
# allowed updates according to simple protonation scheme
# r_min and r_max in nm, 0 ≤ prob ≤ 1
# cut-on and cut-off for distance-based probability
allowed_updates[frozenset(["IM1H", "OAC"])] = {"r_min": 0.140, "r_max":
    0.161, "prob": 0.994}
allowed_updates[frozenset(["IM1", "HOAC"])] = {"r_min": 0.140, "r_max":
    0.154, "prob": 0.098}
allowed_updates[frozenset(["IM1H", "IM1"])] = {"r_min": 0.140, "r_max":
    0.155, "prob": 0.201}
allowed_updates[frozenset(["HOAC", "OAC"])] = {"r_min": 0.140, "r_max":
    0.162, "prob": 0.684}

file_nr: int = int(sys.argv[1])
last_run: int = int(sys.argv[2])

Path("./out").mkdir(parents=True, exist_ok=True)
Path("./traj").mkdir(parents=True, exist_ok=True)
Path("./psf").mkdir(parents=True, exist_ok=True)

psf_file = "final.psf"
if file_nr > 1:
    psf_file = f"psf/npt_{file_nr-1}.psf"
crd_file="final.crd"
psf_for_parameters = "psf_for_parameters.psf"
crd_for_parameters = "crd_for_parameters.crd"
npt_restart_file = "final_npt_7.rst"
PARA_FILES = [
     "toppar_drude_master_protein_2013f_lj04_modhpts_chelpg.str",
     "hoac_d.str",
```

```python
    "im1h_d.str",
    "im1_dummy_d.str",
    "oac_dummy_d.str",
    ]
para_files = [f"../../toppar_dummy/{para_file}" for para_file in
    PARA_FILES]
# option for npT (adds a MonteCarloBarostat) and nVT ensembles
ensemble = "nVT"

# obtain simulation object
simulation = generate_im1h_oac_dummy_system(
    psf_file = psf_file,
    crd_file = crd_file,
    restart_file = npt_restart_file,
    para_files = para_files,
    ensemble = ensemble)
# simulation object with one molecule of each possible species for setting up templates
simulation_for_parameters = generate_im1h_oac_dummy_system(
    psf_file = psf_for_parameters,
    crd_file = crd_for_parameters,
    para_files = para_files)

# get ionic liquid templates
templates = ProtexTemplates([OAC_HOAC, IM1H_IM1], (allowed_updates))

# wrap system in ProtexSystem
ionic_liquid = ProtexSystem(simulation, templates, simulation_for_parameters)

# load names to get the correct protonation states
if file_nr > 1:
    ionic_liquid.load_updates(f"out/updates_{file_nr-1}.txt")
    ionic_liquid.loadState(f"traj/npt_state_{file_nr-1}.rst")
# initialize update method
```

```
to_adapt = [("OAC", 150, frozenset(["IM1H", "OAC"])), ("HOAC", 350,
    frozenset(["IM1", "HOAC"]))]
# option to turn enhancements of update class on/off
update = KeepHUpdate(ionic_liquid, all_forces = True, to_adapt = to_adapt,
    include_equivalent_atom = True, reorient = True)
# initialize state update class
# function for distance based probability:  step (standard), linear or cosine
prob_function = linear
if file_nr == 1:
    state_update = StateUpdate(update, prob_function = prob_function)
else:
    # save and load update with the list of blocked transfers)
    state_update = StateUpdate.load(f"out/state_update_file_nr-1.pkl", update)

dcd_save_freq = 200
state_save_freq = 200
ionic_liquid.simulation.reporters.append(DCDReporter(f"traj/npt_{file_nr}.
    dcd", int(dcd_save_freq)))
ionic_liquid.simulation.reporters.append(
    StateDataReporter(
        sys.stdout,
        state_save_freq,
        step=True,
        time=True,
        potentialEnergy=True,
        kineticEnergy=True,
        totalEnergy=True,
        temperature=True,
        volume=True,
        density=True,
    )
)
```

```
ionic_liquid.simulation.reporters.append(DrudeTemperatureReporter(f"out/drude_temp_{file_nr}.out",
    state_save_freq))
ionic_liquid.simulation.reporters.append(EnergyReporter(f"out/energy_{file_nr}.out", state_save_freq*10))

dt = 0.0005 #ps
time_between_updates = 7 #ps
sim_time = 500  # ps
update_steps: int = 2 #only even numbers
steps_from_update_before_reporter: int = update_steps // 2
steps_from_update_after_reporter: int = update_steps // 2
if update_steps % 2 != 0:
    warnings.warn("Probably working, not entirely tested. To be on the safe side use an even number for
        'update_steps'", UserWarning)
    steps_from_update_after_reporter += 1

steps_between_updates = int(time_between_updates/dt) #int division not working, dt too small
if ((1/dt)*time_between_updates)%(1/dt)!=0:
    time_between_updates = round(steps_between_updates*dt,8)
    msg = f"Not possible to have an integer step value with {time_between_updates}/{dt}! \
            It is rounded down. Hence the effective time between updates is now {steps_between_updates*dt}"
    warnings.warn(msg, UserWarning)

init_steps_between_updates = steps_between_updates-steps_from_update_before_reporter
#remove the steps taken during the updates process
real_steps_between_updates = steps_between_updates-update_steps
final_steps_between_updates = steps_from_update_after_reporter
```

```python

transfer_cycles = sim_time/time_between_updates
total_steps = sim_time/dt
if file_nr == 1:
    actual_transfer_cycles = transfer_cycles -1
    assert total_steps == (init_steps_between_updates +
        (real_steps_between_updates+update_steps)*(actual_transfer_cycles)+final_steps_between_updates)
    simulation.context.setTime(0.)
else:
    actual_transfer_cycles = transfer_cycles
    assert total_steps == (real_steps_between_updates+update_steps)*(actual_transfer_cycles)
    simulation.currentStep = int(total_steps*(file_nr-1)-1)


infos = {"dcd_save_freq": dcd_save_freq, "sim_time(ps)": sim_time, "update_steps": update_steps,
    "steps_between_updates": steps_between_updates, "dt(ps)": dt}
charge_reporter = ChargeReporter(f"out/charge_changes_{file_nr}.out",
    dcd_save_freq, ionic_liquid, header_data=infos)
ionic_liquid.simulation.reporters.append(charge_reporter)

print(f"{init_steps_between_updates=}")
print(f"{update_steps=}")
print(f"{real_steps_between_updates=}")
print(f"{final_steps_between_updates=}")
if file_nr == 1:
    print("First run")
    ionic_liquid.simulation.step(int(init_steps_between_updates))
for step in range(1,int(actual_transfer_cycles+1)):
    print(step)
    state_update.update(int(update_steps))
```

```
    ionic_liquid.simulation.step(int(real_steps_between_updates))
if file_nr == last_run:
    print(f"Last run (Nr. {last_run})")
    ionic_liquid.simulation.step(int(final_steps_between_updates))

# restart files
state = ionic_liquid.simulation.context.getState( getPositions=True, getVelocities=True )
with open(f"traj/npt_{file_nr}.rst", 'w') as f:
    f.write(XmlSerializer.serialize(state))

ionic_liquid.save_updates(f"out/updates_{file_nr}.txt")
ionic_liquid.write_psf("final.psf", f"psf/npt_{file_nr}.psf", psf_for_parameters)
ionic_liquid.saveState(f"traj/npt_state_{file_nr}.rst")
State_update.dump(f"out/state_update_file_nr.pkl")
```

Conductivity

Table 11: Conductivity σ of the pure IL, calculated with different versions of `protex`. (Data for Fig. 10 of the article.)

`protex` version	σ [$\mathrm{S\,m^{-1}}$]	STDEV [$\mathrm{S\,m^{-1}}$]
without `protex`	0.21	0.06
original `protex`	0.28	0.09
1.55 Å	0.31	0.10
closest neighbors	0.43	0.13
RDF first shell	2.07	0.88

Diffusion coefficients

Table 12: Diffusion coefficients D [$10^{-5}\,\mathrm{cm^2\,s^{-1}}$] of each species in the pure IL, calculated with different versions of `protex`. (Data for Fig. 11 of the article.)

`protex` version	Im_1	Im_1H^+	HOAc	OAc^-
without `protex`	0.153 ± 0.002	0.045 ± 0.002	0.104 ± 0.002	0.043 ± 0.002
original `protex`	0.196 ± 0.005	0.056 ± 0.003	0.114 ± 0.003	0.062 ± 0.003
1.55 Å	0.206 ± 0.011	0.060 ± 0.005	0.126 ± 0.006	0.068 ± 0.008
closest neighbors	0.216 ± 0.013	0.073 ± 0.004	0.133 ± 0.010	0.067 ± 0.011
RDF first shell	0.302 ± 0.020	0.117 ± 0.020	0.201 ± 0.027	0.113 ± 0.022

Probability correction

Below we show the time evolution of the number of species separately for each replica with varied strengths of the probability correction.

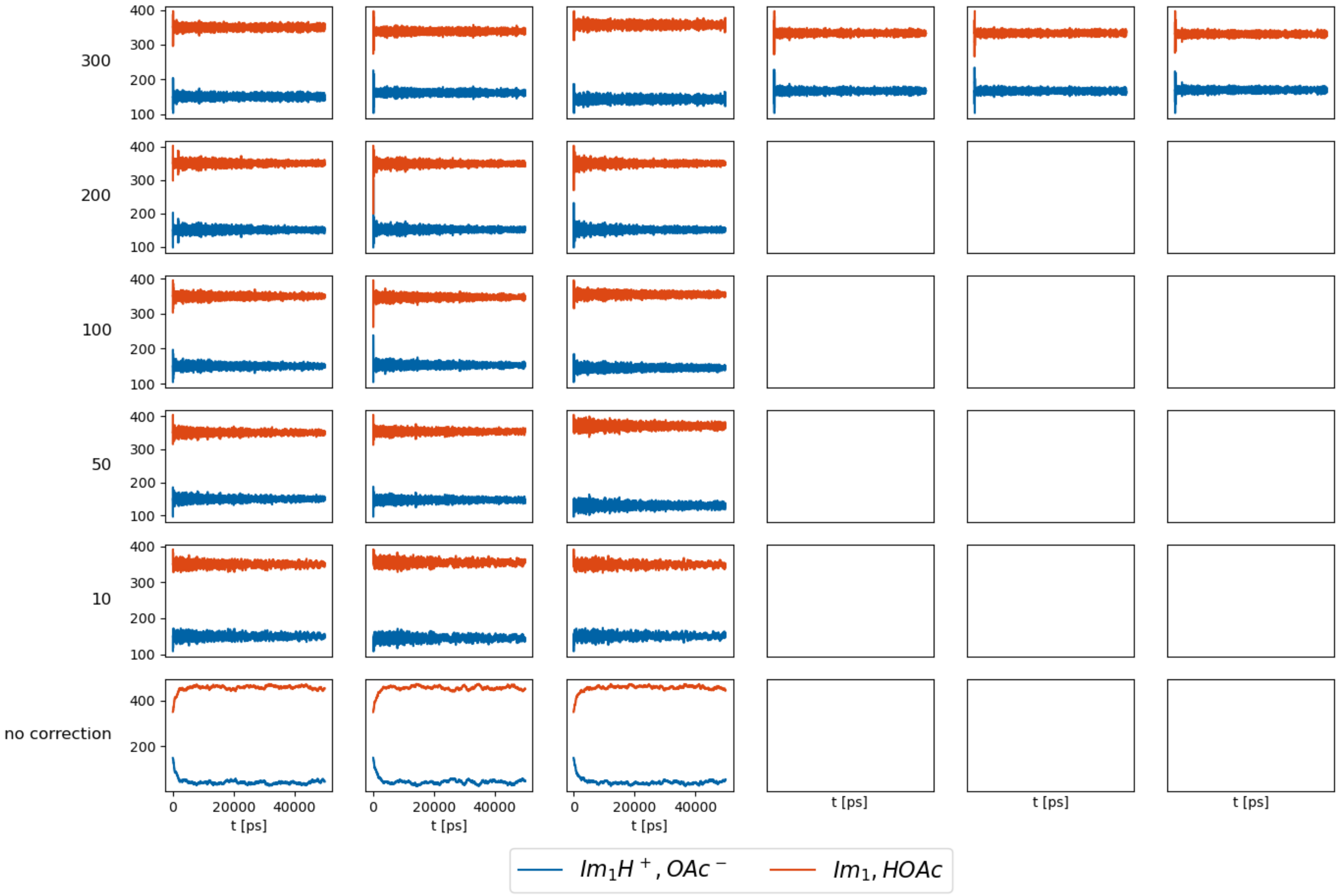

Figure 2: Time evolution of the number of each species with varied probability correction factors. Individual data for each replica. (Raw data for Fig. 8 of the article.)

Density

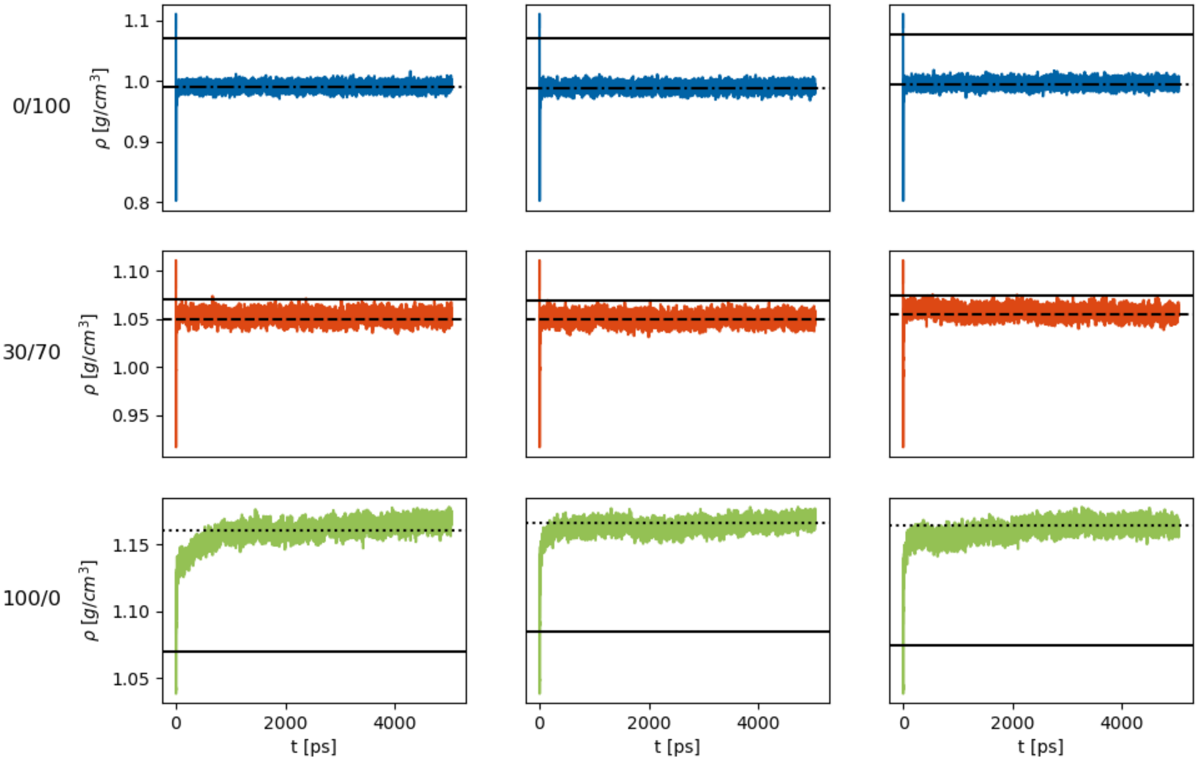

Figure 3: Time evolution of the density of the systems starting from 0 % (blue), 30 % (orange) and 100 % (green) ionic species during equilibration without proton transfers. Individual data for three replicas each. The average values used in the article are shown by the dash-dotted, dashed, and dotted black lines, respectively. The experimental density is shown by the solid black line.

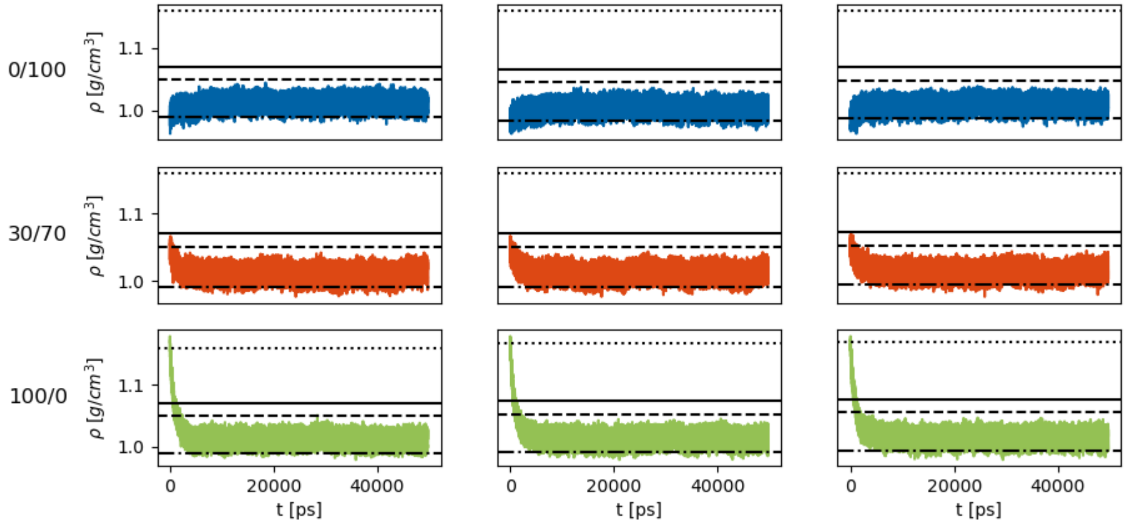

Figure 4: Time evolution of the density of the systems starting from 0 % (blue), 30 % (orange) and 100 % (green) ionic species during simulations with proton transfers. Individual data for each replica without any averaging. The average values used in the article are shown by the dash-dotted, dashed, and dotted black lines, respectively. The experimental density is shown by the solid black line. (Raw data for Fig. 7 of the article.)
